# Ethnomedicinal Plants of Hasankeyf (Batman-Turkey)

**DOI:** 10.3389/fphar.2020.624710

**Published:** 2021-03-11

**Authors:** Yeter Yeşil, İlyas İnal

**Affiliations:** ^1^ Faculty of Pharmacy, Department of Pharmaceutical Botany, Istanbul University, Istanbul, Turkey

**Keywords:** medicinal plants, ethnobotany, ancient city, mesopotamia, anatolia

## Abstract

Hasankeyf is an ancient city, dating back to more than 10,000 years, in the Southeast Anatolia Region of Turkey. The area is separated by the Tigris River on both sides and located in the Batman province. However, as a result of a dam project, in February 2020, this ancient city and some of its surrounding villages were totally flooded. The residents were moved to new settlements. This study aimed to prevent the possible loss of ethnomedicinal knowledge of plants due to migration as well as to pass on this knowledge to the future generations. The field studies were conducted between March 2017 and November 2019 in the city center and 22 rural settlements of Hasankeyf. Also, the areas where intensive migration was experienced were visited frequently. Interviews were conducted with a total of 131 participants (76 women and 55 men) while gathering plants with them. Information was collected through interviews and questionnaires. The results were analyzed by quantitative indices of information consensus factor (FIC) and use value (UV). A total of 94 plant taxa belonging to 40 families were identified in the study area. The most common medicinal plant families are Lamiaceae (13), Asteraceae (8), Rosaceae (6), Malvaceae (6), Amaryllidaceae (5), Brassicaceae (4), and Solanaceae (4). The most common preparations were infusion, fresh application, and crushing. The taxa having the highest count of use value (UV) were *Teucrium polium*, *Matricaria aurea*, *Urtica dioica*, *Mentha longifolia*, and *Quercus brantii*. Besides, the recorded ailments were grouped into categories based on information provided by the interviewees. The most important use categories among the informants were diabetes, gastrointestinal disorders, respiratory disorders, and dermatological disorders. The present study represents the first medical-ethnobotanical documentation and analysis of the traditional use of medicinal plants in Hasankeyf.

## Introduction

Medicinal plants have been used globally throughout the centuries to treat various disorders and ailments. About 80% of the world population uses traditional medicine for primary health care needs (WHO, 1993). More than 50,000 of approximately 422,000 flowering plants in the world are used for medicinal purposes (Hamilton, 2004). The Covid 19 global epidemic has prompted renewed interest in medicinal plants (Vandebroek et al., 2020).

Turkey has a rich flora, with about 11.700 species (Davis, 1965–1985; Davis et al., 1988; Güner et al., 2000; Güner et al., 2012; Güner, 2014; Güner et al., 2018) and an endemism rate of 34% (Güner, 2014). The majority of people who live in rural areas make use of this rich diversity of plants. Systematic ethnobotanical studies only began in the mid-1990s (Ertuğ and Güner, 2014). According to the database of Turkish Folk Medicines Knowledge Base (TUHIB), the number of medicinal plant species in Turkey is more than 1,000 (Yeşilada, 2002; Yeşilada, 2005). In recent years, many studies have been published in Turkey (Günbatan et al., 2016; Korkmaz et al., 2016; Paksoy et al., 2016; Uzun and Kaya, 2016; Baykal and Atamov, 2017; Bulut et al., 2017; Güneş et al., 2017; Karcı et al., 2017; Kartal and Güneş, 2017; Özdemir and Kültür, 2017; Akgül et al., 2018; Sargin and Büyükcengiz, 2018; Nacakçı and Dutkuner, 2018; Gürbüz et al., 2019; Karakaya et al., 2019; Nadiroğlu et al., 2019; Polat, 2019; Çelik and Yeşil, 2020; Güler et al., 2020; Kaya et al., 2020; Kılıç et al., 2020). However, in southeastern Turkey, which covers the study area, only a limited number of ethnobotanical studies have been conducted (Gençay, 2007; Akan et al., 2013; Akgül et al., 2018; Bulut et al., 2019; Yeşil et al., 2019; Yeşil and Inal, 2019; Kılıç et al., 2020).

**FIGURE 1:**
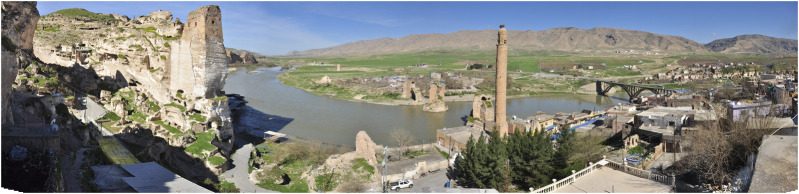
Hasankeyf center with historical construction remains 2013.

**FIGURE 2:**
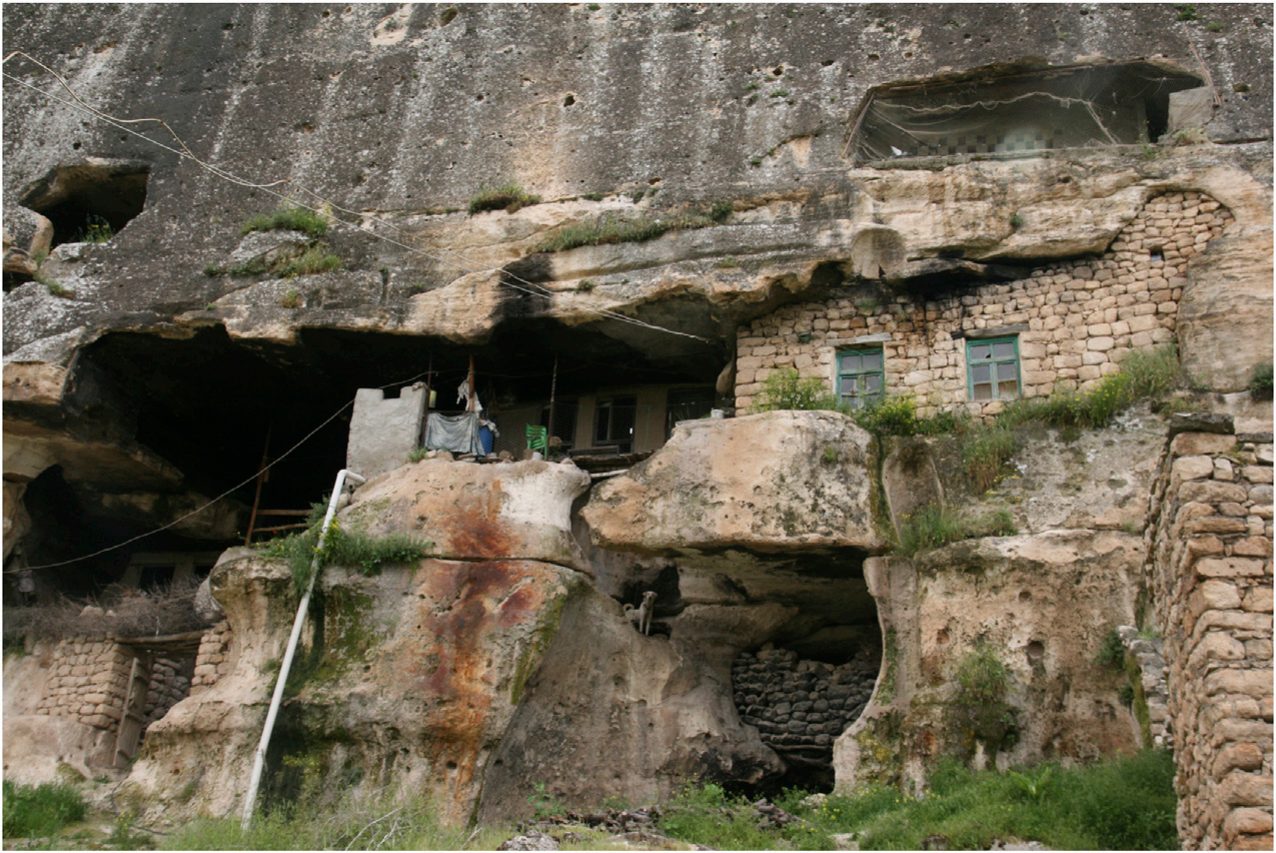
A cave house in the ancient city, 2019.

Hasankeyf is an ancient settlement located in the southeast of Turkey (Batman province) (Figure 1). It is located on the Tigris River in the valley extending from the Persian Gulf, the place of a medieval settlement. Dating back to the Middle Bronze Age, the settlement later became a center on the Silk Road. Life in the region was influenced by various powers from the prehistoric times to the Romans and Selcuks (Ahunday and Balkız, 2009). The name “Hasankeyf” is derived from “kepa, kipas, kefa, kaife,” which means “rock” in Arami or Arameik, Assyrian, Hebrew, Syriac, and Arabic language. The Assyrians named the settlement as “Castrum Kepha” (Rock Castle) during their period of dominance till the 7th century AD (Arık, 2002; Özgen, 2011). Until the 1960s, most of Hasankeyf’s residents lived in ancient caves built into cliffs along the river. Today, some residents still live in the caves (Figure 2).

According to an archaeological study conducted in Hasankeyf Höyük, Hasankeyf was a hunter–gatherer settlement without ceramic ware. So far, the wild plant species found during the excavations in the region include almonds, pistachio, hackberry, lentil, and indeterminate nut species (Miyake et al., 2012). However, most archaeological sites in the region will be submerged by the construction of the Ilısu Dam, which is a part of the Southeast Anatolian Project [Güneydoğu Anadolu Projesi (GAP)], one of Turkey’s largest hydroelectric projects to date (Çevik, 2012; Miyake et al., 2012). The water level of the dam has raised the water level of the Tigris River, which now submerges more than 80% of the ancient city. Seven historical structures were moved to the new settlement. The inhabitants of the city center and three villages lost their land and livelihoods and as a result moved to the new settlement.

In a floristic study conducted in the Hasankeyf district center and its surroundings, 472 taxa belonging to 279 genera and 64 families were identified (Atamov et al., 2014). According to this study, 20 taxa are under threat because they are endemic and found only in this region. In addition, recently two plant species were identified in the sites of the ancient city and the Ilısu Dam: *Onopordum hasankeyfense* Pınar and Behçet (Pınar and Behçet, 2014) and *Salvia hasankeyfense* Dirmenci, Celep, and O. Güner (Celep et al., 2015). Populations of these species are at risk of extinction if conservation measures are not taken, and the species are in the category of Critically Endangered (CR) species.

In addition to historic places, plants, and other living things, traditional knowledge is also under threat of being lost in the research area due to the dam project. It is inevitable that ethnobotanical knowledge would be forgotten over time, especially due to the migration. It is therefore vital to conduct a detailed investigative research on the traditional ethnomedicinal knowledge of plants used in Hasankeyf and its surroundings. In our project design, we focused on: (1) compiling the ethnomedicinal knowledge of plants in the old and multicultural district; (2) frequently visiting the areas where intensive migration will be experienced due to the dam (Hasankeyf center and some villages); (3) spending sufficient time with the local people to obtain more intensive ethnobotanical knowledge; (4) comparing the obtained ethnomedicinal data with those of nearby regions; and (5) uncovering new ethnomedicinal uses of plants.

## Materials and Methods

### The Study Area

**FIGURE 3:**
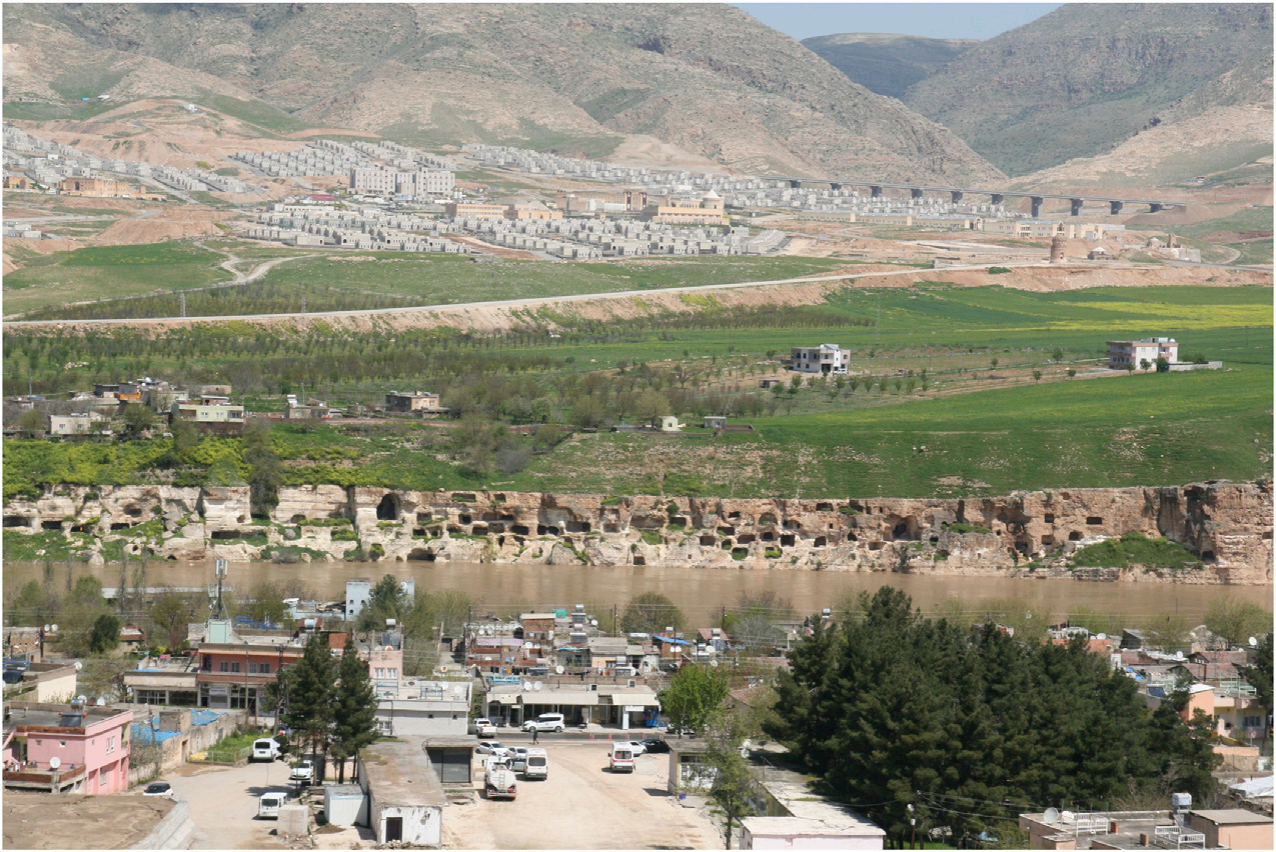
The old and new settelements April 2019.

Hasankeyf is located to the north of the Midyat and Raman hills (Özgen, 2011). The old city center was located 37 km Southeast of Batman city and generally had low lying hills (Özgen, 2011). The new settlement is located at the opposite slope of the old city, on the other side of the Tigris River (Figure 3).

**FIGURE 4:**
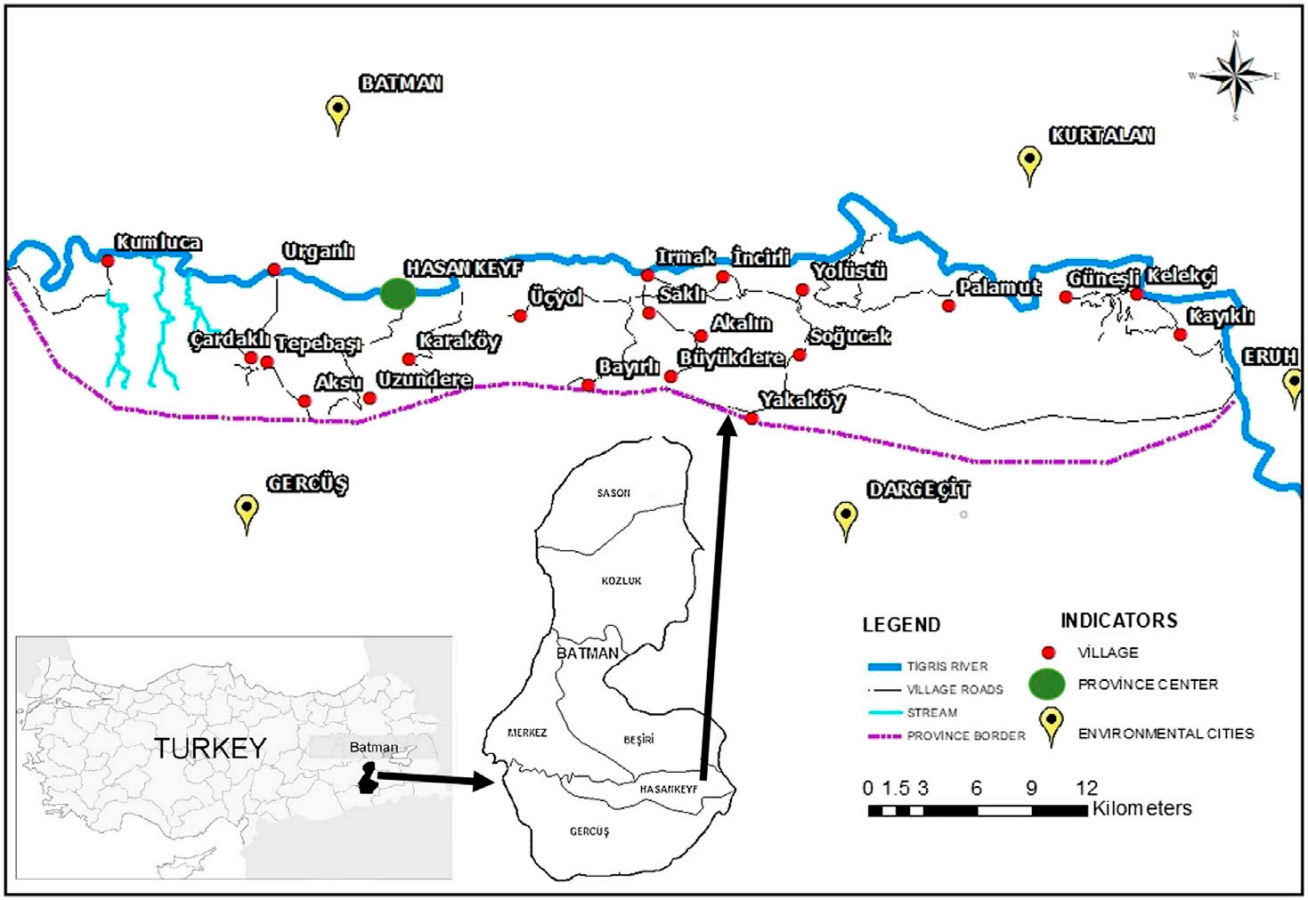
The map of the study area, and its location in Batman Province and in Turkey.

**FIGURE 5:**
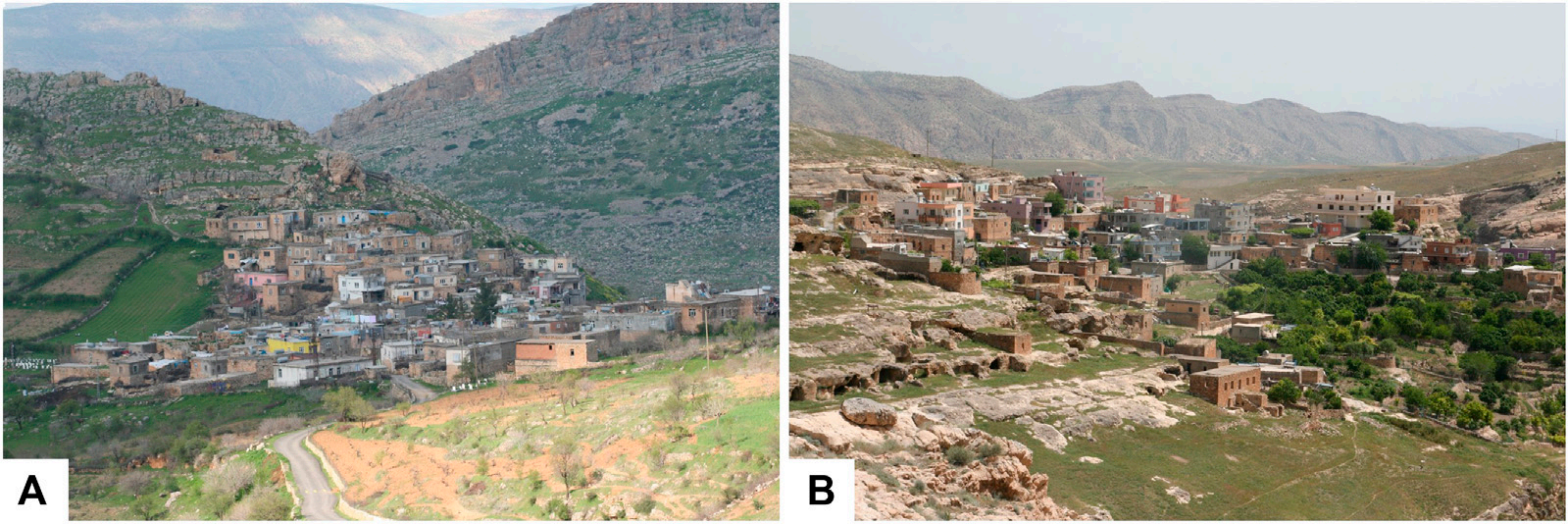
Two villages of Hasankeyf **(A)** Karaköy **(B)** Üçyol.

The Hasankeyf region has an area of 529.95 sq. km. There were 22 villages (Akalın, Aksu, Bayırlı, Büyükdere, Çardaklı, Gaziler, Güneşli, Irmak, İncirli, Karaköy, Kayıklı, Kelekçi, Kumluca, Öğütlü, Palamut, Saklı, Soğucak, Tepebaşı, Uzundere, Üçyol, Yakaköy, and Yolüstü) in rural areas and three settlements (Bahçelievler, Kale, and Eyyubi) in urban (Figures 4, 5); however, three settlements of the city center and three villages were flooded. The altitude is between 520 and 1,200 m. The Tigris River has an impact on the climate of the region, thus contributing to the area’s mild winters (Batman Governorship, 2018). The annual average temperature is 25°C. The highest average temperature is 40–43°C (July), while the lowest average temperature is 6–8°C (January). Precipitation primarily occurs in winter and spring, with an average annual rainfall of about 542 mm (Climate data for cities worldwide, 2012). This area belongs to the Irano–Turanian Plant Geography Region and falls within the C8 grid square according to the grid classification system developed by Davis (1965–1985). The dominant vegetation is steppe. There are small oak forests and maquis at an altitude of 800 m and above. The area is covered with small oak forests and maquis. *Amygdalus orientalis*, *Amygdalus arabica* Oliv., *Celtis tournefortii*, *Crataegus azarolus* L., *Olea europaea* Lindl., *Pistacia* species, *Paliurus spina-christi*, and *Juniperus oxycedrus* are among the important maquis species in this area (Atamov et al., 2014).

Since it was an ancient city, the main source of livelihood of Hasankeyf was tourism in the city center. On the other hand, agriculture, horticulture, and livestock production were the main sources of income in the villages. Wheat is the main product of Hasankeyf, in addition to grape, almond, fig, lentil, and watermelon (Batman Governorship, 2018).

### Interviews With Local People

A questionnaire was administered to the local people through face-to-face interviews (Appendix A).

The interviews were conducted with people in their homes, mosques, village squares, teahouses, gardens, or fields. Also, villages that would be submerged were visited more frequently. The official language in the study area is Turkish; however, most of the locals speak Arabic and Kurdish in Hasankeyf center, while the people who live in the villages generally speak Kurdish. The interviews were conducted in their mother tongues to ensure that they are able to express themselves comfortably. In the first year (2017), field studies were conducted by both authors. In the following 2 years (2018–2019), YY continued to study in the villages with the assistance of a local guide who speaks Kurdish and Turkish, and the interviews were conducted in Kurdish. At the town center, from the beginning of the study, the interviews were conducted in Arabic, Kurdish, and Turkish with the assistance of a local guide who can speak Arabic, Kurdish, and Turkish. For this reason, there was no problem of communication in the study area.

The Code of Ethics of the International Society of International Society of Ethnobiology (2008) was followed. The purpose of the study was explained to all participants and interviews were held only after they gave their approval to participate. Following the rules of Arabic and Kurdish language, local plant names were given in Latin alphabets.

### Plant Materials

The field studies took place from March 2017 to November 2019. During this time, 171 plant specimens were collected. The plants were identified using the Flora of Turkey and the East Egean Islands (Davis, 1965–1985; Davis et al., 1988; Güner et al., 2000), A checklist of the flora of Turkey (vascular plants) (Güner et al., 2012), Illustrated flora of Turkey [vol. 1 (Güner, 2014) and vol. 2 (Güner et al., 2018)]. Plant specimens were deposited at the Herbarium of Istanbul University’s Faculty of Pharmacy (ISTE). Plant parts obtained during the field studies are numbered with HSF (Hasankeyf) code and stored in envelopes and jars. The scientific names of the plant taxa were checked and controlled according to the checklist of the flora of Turkey (Güner et al., 2012) and The Plant List website (The Plant List, 2013).

### Ethnobotanical Indices

#### Informant Consensus Factor (FIC)

The FIC was originally developed by Trotter and Logan (1986) and then readapted by Heinrich (2000). The FIC value was used to analyze whether there was a consensus amongst the informants of the study area in the use of plants for various ailment categories. The FIC was calculated by the following formula: FIC = Nur − Nt/Nur − 1, where Nur refers to the number of use citations in each category and Nt to the number of the species used. This measures the homogeneity of the ethnobotanical knowledge. FIC values range from 0 to 1. A high value indicates that informants are in agreement on the use of taxa for a category of illness. A low value indicates that plants are chosen randomly or informants do not exchange information about their uses. A high FIC indicates the informants’ agreement about the taxa used for the treatment of ailments of a certain use category (Table 1).

**TABLE 1:** The categories of ailments and associated informant consensus factor (FIC) values.

The ailment categories	Number of taxa	Use citations	FIC
Internal and external inflammatory problems	13	101	0.89
Diabetes	12	64	0.82
Urogenital and kidney problems	15	69	0.79
Respiratory diseases	29	98	0.71
Rheumatic pain	5	13	0.66
Ear disorders	6	15	0.64
Blood problems: hypertension, hematinic, hemostatic	7	17	0.62
Hemorrhoids	6	14	0.61
Cardiac diseases	3	6	0.60
Gastrointestinal disorders	40	97	0.59
Snake bite, scorpion sting, fly bite, and parasitic disease	4	8	0.57
Healing wounds, skin and hair care	11	25	0.58
Anticancer	3	5	0.50
Boosting the immune system	3	5	0.50
Toothache	6	12	0.45
Delighting, sedative, regulate the taste of the mouth	5	8	0.42
Eye disorders	7	11	0.40

#### Use Value (UV)

The relative importance of each species that the informants provided information for was found using the UV calculation. UV was calculated based on the following formula (Phillips et al., 1994):

$$UV=\sum \frac{U}{N}$$

Here, UV is the use-value of a species; U is the number of uses cited by each informant for a given plant species and *N* is the total number of informants interviewed for a given plant. If the UV value is low, it indicates that the plant is not widely known. However, if the UV value is high, IT suggests that the plant is a frequently used, popular plant.

#### Jaccard’s Similarity Index

A Venn diagram was drawn based on the medicinal taxa by the three selected regions and the related Jaccard Similarity Index for each pairing of the considered regions was calculated.

Jaccard’s similarity index considers the similarity between two OTUs (operational taxonomic units) as the number of attributes shared divided by the total number of attributes present in either of the OTUs. Jaccard’s index may be expressed as follows:

J=C/A+B

Here, A is the number of attributes present in OTU a, B is the number of attributes present in OTU b, and C is the number of attributes present in both OTUs a and b. The number of attributes present in either of the OTUs is given by A + B (Jaccard, 1908).

## Results

### Demographic Features of the Interviewed Informants

We interviewed a total of 131 informants, 76 females (58.01%) and 55 males (41.98%), whose age ranged from 8 to 98 years. Their education levels were varying—33.58% having no education, 38.93% having primary education, 19.08% having secondary education, and only 8.39% having tertiary education. Most of them were Kurdish; thus, interviews with this group of informants were conducted in Kurdish and sometimes in Turkish. The female informants were less educated than males—26.71% of female informants had not attended school, while only 6.87% of male informants had not attended school. Moreover, females aged 55 and over were not educated at all, especially those in rural areas. The demographic details of the informants are summarized in Table 2.

**TABLE 2:** Demographic details of the interviewed informants.

Categories	Subcategories	Number of informants	
Gender		Female	Male
		76	55
Age	8–21	1	3
	21–30	8	4
	31–40	13	9
	41–50	14	9
	51–60	17	12
	61–70	19	12
	71≥	4	6
Education level	None	35	9
	Primary	28	23
	Secondary	10	15
	Tertiary	3	8
Informant status	Villages	66	36
	City center	10	19
Employment status	Retirement	—	8
	Farmer	20	34
	Housewife	51	—
	Unemployed	1	4
	Other jobs	4	9
Total		131	

### General Figures and Most Represented Families and Species

**FIGURE 6:**
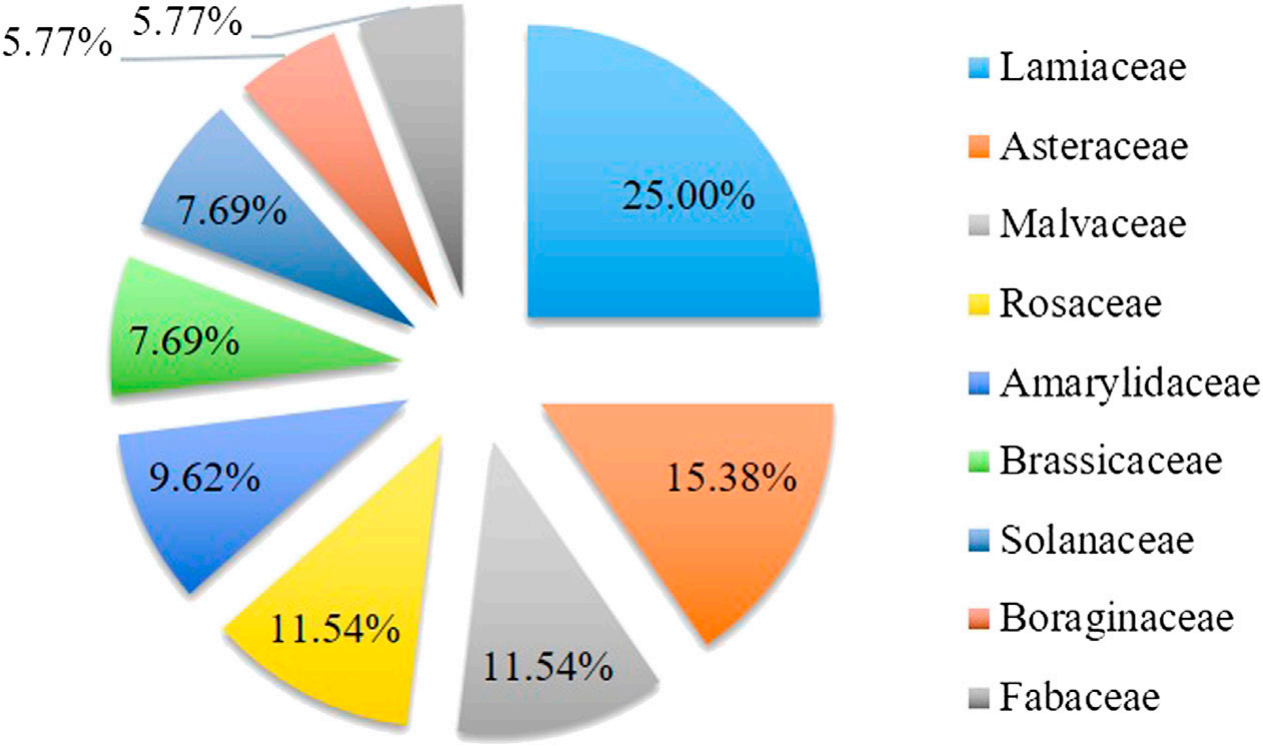
The percentages of most used plant families in Hasankeyf.

The plants utilized for medicinal purposes in the Hasankeyf district are listed in Table 3 and arranged in alphabetical order of their family and botanical names. In the course of this study, 171 specimens were collected and 94 taxa belonging to 72 genera and 40 families were recorded. Twenty families were represented by just one taxon, while the other 20 families were represented by 2 or more taxa. The predominant families were Lamiaceae (13), Asteraceae (8), Malvaceae (6), Rosaceae (6), Amaryllidaceae (5), Brassicaceae (4), Solanaceae (4), Boraginaceae (3), and Fabaceae (3) (Figure 6).

**TABLE 3:** Ethnomedicinal usage of the plants in Hasankeyf.

Family	Botanical name, herbarium, or collector number	Local name	Plant part (s) used	Preparation	Utilization method	Therapeutic effect/ailments treated	UV
Adiantaceae	*Adiantum capillus-veneris* L. ISTE 116163	Pore fatme (K), şaar, cıbbar (A)	Aerial parts	Infusion	Internal	Diuretic, abdominal pain, antitussive, cardiac diseases, shortness of breath	0.03
Amaryllidaceae	*Allium ampeloprasum* L. ISTE 116272	Sîrik, sîrim, sîrikapenir (K)	Leaves Bulbs	Fresh	Eaten External	Antihypertensive Eye burning	0.04
	^a^ *Allium cepa* L.	Basal (A), pîvaz (K)	Bulb	Fresh Fresh Onion juice with soap, direct	Eaten External Smelling Dropped into the ear	Galactagogue, Removing inflammations caused by plant spines on the skin and removing the spines Respiratory tract Ear pain	0.29
	*Allium kharputense* Freyn and Sint. ISTE 115344	Sîrim, surım, solyask, sorelask (K)	Bulb, leaves	Crushed	External (to the eyelids)	Eye pain	0.05
	^a^ *Allium sativum* L.	Sîr (K), ŝum, fum (A)	Bulbils	Fresh With salt Added to olive oil	Eaten External Internal [1 × (1)2, mh] External	Eye diseases, antihypertensive, Toothache Stomachache Alopecia	0.21
	*Allium scorodoprasum* L. ISTE 116125	Sîrik, sîrim, sîrimapenir (K)	Bulbils, leaves	Crushed	External	Eye burning	0.03
Anacardiaceae	*Pistacia palaestina* Boiss. ISTE 115361	Bıttım (A), qezwan (K)	Fruits	Crushed	Prepared soap External	Hair loss, hair care, skin care	0.17
	*Pistacia eurycarpa* Yalt. ISTE 115361	Bıttım (A), qezwan, benik (K)	Resin Fruits	Chewing Crushed	Internal As coffee	Lung and liver diseases Delighting	0.16
	*Rhus coriaria* L. ISTE 115365	Sumaq, sımaq (A, K), tirş (K)	Fruits	Infusion	Gargle Internal	Mouth sore Antidiarrheic, antiemetic, digestion problems	0.23
Apiaceae	^a^ *Pimpinella anisum* L. HSF27	Elison (K)	Fruits	Infusion	Internal	Carminative (children)	0.08
	*Scandix stellata* Banks and Sol. ISTE 115038	Ziçirk (K)	Aerial parts	Fresh	Eaten	Digestion troubles	0.03
Araceae	*Arum rupicola* Boiss. ISTE115336	Kardî (K), nubê (A)	Leaves	Cooked, dried then cooked	Eaten	Antitussive, menstrual pain	0.17
	*Biarum* sp. HSF19	Lopka kocabatri (K)	Dried underground parts	Powdered	Internal (1 ts)	Antidiarrheic, hemorrhoids	0.04
Asteraceae	*Achillea aleppica* DC. ISTE 117182	Kulilka maran, kulilka zer (K)	Aerial parts	Infusion	Internal (1 × 1/2, h)	Stomachache	0.06
	*Achillea arabica* Kotschy ISTE117183	Kulilka maran, kulilka zer (K)	Aerial parts	Infusion	Internal (1 × 1/2, h)	Stomachache	0.08
	*Chondrilla juncea* L. ISTE 116120	Giyayêbenuşta (K)	Latex of root	Fresh	Eaten	Liver troubles	0.01
	*Cota altissima* (L.) J. Gay ISTE 116117	Beybanuç, beybun (A, K)	Inflorescences	Infusion	Internal	Abdominal pain, common cold, antitussive	0.08
	*Cota austriaca* (Jacq.) Sch. Bip. ISTE 116150	Beybanuç, beybun (A, K)	Aerial parts	Decoction	Internal	Abdominal pain, common cold, antitussive	0,08
	*Cota tinctoria* (L.) J. Gay ISTE 115673	Giyayêzer (K)	Aerial parts	Infusion	Internal [1 × (1)2, h]	Cardiac diseases, shortness of breath	0.09
	*Echinops orientalis* Trautv. ISTE 116276	Serteşik, sitrik (K)	Aerial parts Inflorescences	Direct	External (prick)	Bloodletting	0.02
	*Matricaria aurea* (Loefl.) Sch. Bip. ISTE 115341	Beybanuç (A,K), giyayêseva, gihaseva (K)	Aerial parts	Decoction	Internal [1 × (1)2, h]	Abdominal pain, menstrual pain, antitussive (two times a day), afterpains, common cold, antitussive	0.44
Boraginaceae	*Anchusa azurea* Mill. ISTE 115383	Gûriz (A, K)	Aerial parts Leaves Flowers	Crushed Sucked	External	Snake bite Regulate the taste of the mouth	0.22
	*Anchusa strigosa* Banks and Sol. ISTE 115371	Gûriz (A, K)	Leaves	Infusion	Internal	Painkiller	0.24
	*Cerinthe minor* L. ISTE 115395	Gayebej, gobelk (K)	Aerial parts	Infusion	Internal	Delighting	0.007
Brassicaceae	*Lepidium draba* L. ISTE115376	Xardal, qinêbêr, qunêberk (K), xerdıl (A)	Aerial parts	Infusion	Internal	Vomitive (children), abdominal pain	0.17
	*Nasturtium officinale* R. Br. ISTE 115436	Tuzik, tüzik, Tûzîk (K)	Aerial parts	Fresh	Eaten	Appetizing, abdominal pain	0.06
	*Sinapis alba* L. ISTE115438	Xerdel, xerdal (K)	Aerial parts	Infusion	Internal	Vomitive (children)	0.08
	*Sinapis arvensis* L. ISTE 116285	Xerdel, xerdal (K)	Aerial parts	Infusion	Internal	Vomitive (children)	0.06
Cannabaceae	*Celtis tournefortii* Lam. ISTE 116195	Gengeres (A), taew, taav (K)	Fruits	Crushed then added to grape molasses Crushed or fresh	Eaten Eaten	Boosting the immune system Abdominal pain	0.01
Capparaceae	*Capparis sicula* Veill. ISTE 115419	Kember, kemberok, kemberok, kember, inok (K), şefellk, welleh (A)	Leaves Buds	Infusion Crashed	Internal External	Antitussive, stomachache Rheumatism, hand injuries, foot pain	0.08
Caryophyllaceae	*Gypsophila pallida* Stapf ISTE 116119	Giyayêhelavê (K)	Roots	Powdered and added to grape molasses	Eaten	Boosting the immune system	0.01
Cucurbitaceae	*Bryonia aspera* Stev. ex Ledeb. ISTE 116189	Xezirvik, xezrowîk, xerzukrivi (K)	Fruit	Fresh	Eaten	Diabetes	0.11
	^a^ *Citrullus lanatus* (Thunb.) Matsum. and Nakai	Zebeş (K)	Bark of fruits	Dried and powdered	Internal	Abdominal pain	0.06
Cupressaceae	*Juniperus oxycedrus* L. ISTE 116155	Hêvrist (K)	Branches	Burned and smoke	Inhalation	Respiratory disorders	0.06
Convolvulaceae	*Cuscuta babylonica* Aucher ex Choisy ISTE 116177	Iqşut (A, K)	Aerial parts	Decoction	Internal	Cardiac diseases	0.02
Dioscoreaceae	*Dioscorea communis* L. ISTE 115421	Darhablelek, derheblenek (K)	Fruits Fruits Underground parts	Burned, Powdered and added water Fresh	Inhalation as frankincense Dropped into the ear (1 ts) External	Toothache Ear pain Arthralgia, rheumatism	0.09
Euphorbiaceae	*Euphorbia craspedia* Boiss. ISTE 115404	Xulişîrik (K)	Latex	Yogurt yeast Direct	Eaten (yogurt) External	Constipation Psoriasis, dermatophyte, toothache	0.15
	*Euphorbia macroclada* Boiss. ISTE 116165	Xulişîrik (K)	Latex	Yogurt yeast Direct	Yogurt eaten External	Constipation Psoriasis, dermatophyte, toothache	0.12
Fabaceae	^a^ *Cicer arietinum* L. HSF15	Nakareş (K), hımmısısut (A)	Seeds	Decoction	Internal	Antidiarrheic, wormer	0.10
	*Glycyrrhiza glabra* L. ISTE 115667	Sûs, sus (A, K)	Roots	Maceration	Internal Internal (1 × 1, mh)	Wormer, stomachache, abdominal pain, Against nausea	0.13
	*Prosopis farcta* (Banks and Sol.) J.F. Macbr. ISTE 116175	Xarnuf (A), xurnuf (K)	Fruits, seeds	Decoction	Internal	Diabetes, antihypertensive	0.09

Fagaceae	*Quercus brantii* Lindl. ISTE 115423	Balot (A), baru, beru (K)	Fruits Leaves	Cooking in embers or fresh Infusion	Eaten [1 × 3 (4)] Internal	Diabetes Diabetes	0.34
	*Quercus libani* Oliv. ISTE 115422	Balot (A), baru, beru (K)	Fruits Leaves	Fresh Infusion	Eaten (1 × 3, h) Internal	Diabetes Diabetes, antitussive, common cold	0.09
Hypericaceae	*Hypericum triquetrifolium* Turra ISTE 115426	Botav (K)	Aerial parts Flowers	Infusion Direct	— External (1 × 1, n)	Liver disorders, diabetes Eye contours, eye pain	0.25
Lamiaceae	*Cyclotrichium leucotrichus* (Stapf ex Rech. F.) Leblebici ISTE 116168	Rehan cebel (A)	Aerial parts	Infusion	Internal	Antitussive	0.03
	*Melissa officinalis* L. ISTE 116136	Giyakî çolê, giyaye tirş (K)	Dried or fresh leaves Aerial parts	Infusion	Internal Internal (1 × 1, 1–2 tgs)	Sedative Headache, respiratory disorders, abdominal pain	0.06
	*Mentha longifolia* (L.) L. subsp. *typhides* (Briq.) Harley ISTE 116164	Pûng (K), pûnge (A)	Dried or fresh leaves Leaves	Infusion Fresh	External (1 × 1) Eaten	Abdominal pain, shortness of breath, halitosis, antitussive, common cold, menstrual disorders, infertility Rheumatism Halitosis	0.37
	*Origanum vulgare* L. subsp. *gracile* (C. Koch) Letsw. ISTE 116162	Rehan cebel (A), rehan (K)	Aerial parts	Infusion	Internal	Antitussives	0.09
	*Salvia multicaulis* Vahl. ISTE 115391	Rihan, giyaçaye, Çaye gahye, siraketin (K)	Fruits	Infusion	Internal	Urinary problems, abdominal pain, menstrual pain, bronchitis	0.19
	*Salvia palaestina* Benth. ISTE 116158	Giyaçaye (K)	Aerial parts	Infusion	Internal	Abdominal pain, menstrual pain	0.08
	*Scutellaria orientalis* L. subsp. *bornmuelleri* (Hausskn. Ex Bornm.) Edmondson ISTE 116124	Giyatâl (K)	Aerial parts	Dried, crush added sugar	Internal (1 × 1, ts, h)	Diabetes	0.03
	*Sideritis libanotica* Labill. ISTE117181	Qirşika şin, şirtik, şirtika şin, giyayê hêjîre (K)	Aerial parts	Infusion	As tea	Abdominal pain, diabetes	0.10
	*Teucrium chamaedrys* L. subsp*. sinuatum* (Celak.) Rech. f. ISTE 115393	Giyatâl (K)	Aerial parts, leaves Leaves	Infusion	Internal (1 × 1) Internal (1 × w)	Diabetes, postnatal drip Diabetes, urinary problems	0.03
	*Teucrium polium* L. ISTE 116130	Bojdank, giyatâl (K), cede (A)	Aerial parts	Infusion Decoction Dried, powdered Boiled, as paste	Internal (1 × 1) Internal (1 × 1, ts) Internal (1 ts, mh) External	Diabetes, hemorrhoids Abdominal pain, Diabetes, urinary problems Eye pain	0.47
	*Thymbra sintenisii* Bornm. and Azn. ISTE 116194	Cahter, catir (K), zahter (A)	Aerial parts	Infusion	Internal (1 × 1)	Afterpains, antitussive, common cold, flu, abdominal pain, stomachache	0.19
	*Thymbra spicata* L. ISTE 116206	Cahter (K), zahter (A)	Aerial parts	Infusion	Internal (1 × 1)	Afterpains, antitussive, common cold, flu, abdominal pain, stomachache	0.17
	*Thymus kotschyanus* Boiss. and Hohen. ISTE 116205	Cahter (K)	Leaves	Infusion	Internal (1 × 1)	Abdominal pain, afterpains, antitussive, common cold, flu, stomachache, throat ache	0.06
Lythraceae	^a^ *Punica granatum* L. ISTE 116133	Hînar (K), rımman (A)	Flowers	Crushed	Eaten (mh)	Hemorrhoids, stomachache	0.12
Malvaceae	*Alcea setosa* (Boiss.) Alef.ISTE 115363	Hîro (K), xıtmi (A)	Aerial parts Roots Aerial parts	Dried and powdered added to bath water Dried and crushed parts with milk	External Internal	Hair care, skin disorders Throat ache, antitussive, bronchitis	0.28
	*Alcea digitata* (Boiss.) Alef. ISTE 116148	Hîro (K), xıtmi (A)	Aerial parts	Infusion of dried and crushed parts with milk	Internal (mh)	Throat ache, antitussive, bronchitis	0.10
	*Althaea cannabina* L. ISTE 116157	Hîro (K), xıtmi (A)	Aerial parts	Infusion of dried and crushed parts with milk Infusion of dried and crushed parts with milk	Internal (mh) Internal	Throat ache Antitussive, bronchitis	0.11
	*Malva neglecta* Wallr. ISTE 115390	Tolık, tolik (K), tolıkê (A)	Aerial parts	Decoction	Internal	Abdominal pain, common cold	0.25
	*Malva nicaeensis* All. ISTE 115412	Tolık, tolik (K), tolıkê (A)	Aerial parts	Decoction	Internal	Abdominal pain, common cold	0.18
	^a^ *Tilia cordata* Mill. HSF10	Ixlamur (A, K)	Inflorescences	Decoction, infusion	Internal	Throat ache, antitussive, bronchitis	0.21
Moraceae	^a^ *Ficus carica* L. subsp. *carica* ISTE 116199	Hêjîr, hejîr (K), tinê (A)	Leaves Fruit Latex Leaves	Decoction Fresh Fresh	External Eaten External	Haemorrhoids, wart Intestinal disorders Toothache Eye pain	0.19
	^a^ *Morus nigra* L. ISTE 116149	Tuye reş, tuye şemme (K)	Fruits	Fresh	Eaten (h) Eaten	Wormer Anticancer, hematinic	0.06
Oleaceae	^a^ *Olea europaea* L. HSF14	Zaytun (A), zeytûn (K)	Leaves	Infusion	Internal (1 × 1, mh)	To defuse	0.05
Papaveraceae	*Papaver glaucum* Boiss. and Hausskn. ISTE 115338	Xicxicok, xacxacok, xecxecoka şehika (K)	Aerial parts	Crashed, as paste	External	Inflamed wound, abdominal pain	0.10
	*Papaver macrostomum* Boiss. and A. Huet ISTE 115082	Xicxicok, xacxacok, xecxecoka pîrçika (K)	Aerial parts	Crushed, as paste	External	Inflamed wound, abdominal pain	0.06
Plantaginaceae	*Plantago major* L. ISTE 117180	Palhavez, pelhevez (K)	Leaves	Fresh	External	Inflamed wound, Removing inflammations caused by plant spines on the skin and removing the spines	0.15
Platanaceae	^a^ *Platanus orientalis* L.	Çinar (K)	Leaves	Infusion	Internal (1 × 2)	Rheumatism, arthrolith	0.07
Poaceae	^a^ *Hordeum vulgare* L. ISTE 115429	Ca, ceh (K)	Fruits	Decoction	Internal	Pass a kidney stone, diuretic	0.08
Portulacaceae	^a^ *Portulaca oleracea* L. ISTE 116209	Parparik pirpar (K), pırperê (A)	Aerial parts	Fresh	Eaten	Constipation	0.05
Ranunculaceae	*Ranunculus cornutus* DC. ISTE 115416	Kunamella (K)	Aerial parts	Fresh, crushed	External	Inflamed wound	0.13
	*Ranunculus millefolius* Banks and Sol. ISTE 116144	Kulilke zer (K)	Aerial parts	Crushed	External	Inflamed wound	0.09
Rhamnaceae	*Paliurus spina-christi* Mill. ISTE 115346	Driyesor, stiri (K)	Fruits Branches	Crushed, added to milk Kept on fair, flowing sap	Internal External	Antitussive Toothache	0.06
Rosaceae	^a^ *Amygdalus communis* L. ISTE 115435	Behîve tal, behîve tal (K)	Fruits	Crushed	External	Mouth sore (children)	0.14
	*Prunus orientalis* (Mill.) Koehne ISTE 116185	Behîva eşk (K)	Fruits	Fresh	Eaten	Diabetes	0.03
	^a^ *Cydonia oblonga* Mill.	Beh (K)	Fruits	Fresh	Inhalation	Sedative	0.007
	^a^ *Persica vulgaris* Mill. (L.) Batsch	Xox (K)	Seeds Leaves	Powdered then added to breast milk Infusion	Dropped into the ear (1 × 2, 3 drs) Dropped into the ear [1 × (2)3, 3 drs]	Ear pain Ear pain	0.03
	*Rosa canina* L. ISTE 116128	Gulşîlan, gulşilav (K)	Flowers Fruits	Infusion Decoction	Internal Internal	Constipation Abdominal pain, antitussive	0.17
	*Rubus sanctus* Schreb. ISTE 115439	Awsâç (A), dirîreşk, dirîreşik, turêşk (K)	Fruits	Fresh	Eaten	Renal disorders hematinic	0.08
Salicaceae	^a^ *Salix alba* L. ISTE 116126	Bî (K)	Leaves Branches, barks	Decoction Infusion	Internal	Antitussive Antitussive, common cold, flu	0.09
Scrophulariaceae	*Verbascum* sp. ISTE 115420	Balûtkefir (A)	Leaves	Crashed	External	Inflamed wound	0.08
Solanaceae	*Hyoscyamus albus* L. ISTE 115364	Benc, penc, tûtinkosa, tûtinkasa (K)	Seeds Leaves	Powdered then added to breast milk Fresh or powdered Crashed Infusion	Dropped into the ear External External Internal	Ear pain Inflamed wound, to remove inflammation caused by spines in the skin and to remove the spines, athlete’s foot, acne Eye redness Stomachache	0.20
	^a^ *Lycopersicon esculentum* Mill.	Balcana sor (K)	Fruits	Fresh	External	Fly bite, scorpion sting	0.03
	*Solanum americanum* Mill. ISTE 116154	Kulike benav (K)	Fruits	Dried, powdered, burned	Keeping the ear over the smoke	Ear problems	0.03
	^a^ *Solanum tuberosum* L.	Kartol (K)	Tubers	Planed and filtered juice	Internal (1 × 1, mh)	Stomachache	0.03
Theaceae	^a^ *Camellia sinensis* (L.) Kuntze	Çay (T)	Leaves	Infusion, added yogurt	Internal	Antidiarrheic	0.08
Urticaceae	*Urtica dioica* L. ISTE 115408	Gezgezok, gezo, gezgezk (K), qırrez (A)	Aerial parts	Fresh Infusion Fresh Fresh	External Internal (1 × 3) External External	Rheumatism, itching Anticancer, anti-inflammatory Hair loss Hemostatic	0.39
	*Urtica pilulifera* L. ISTE 115339	Gezgezok, gezo, gezgezk (K), qırrez (A)	Whole parts Aerial parts	Fresh Infusion with fruit of *R. coriaria*	External Internal (1 × 3) External	Anti-inflammatory, rheumatism, itching Diabetes, anticancer, antitussive Hemostatic	0.07
Vitaceae	^a^ *Vitis vinifera* L. ISTE 115381	Anup (A), mêw, tirîyi mezruni (K)	Fruits	Molasses	Eaten	Boosting the immune system, antitussive	0.04
Zygophyllaceae	*Tribulus terrestris* L. ISTE 117179	Seddinank, seddirank (K)	Aerial parts	Infusion	Internal (1 × 2)	Diabetes, stomach disorders, urinary disorders	0.22
Stereocaulaceae	*Leptogium* sp. HSF20	Hinatirk, hennatitîk, hınatitk (K)	Whole parts	Powder	External	Dying hairs and hands, against diaper rash, inflammation, dermatophyte	0.23

^a^ Cultivated plants.

Plant numbers: HSF, Hasankeyf; ISTE, Herbarium of Istanbul University’s Faculty of Pharmacy.

Local names: A, Arabic; K, Kurdish; T, Turkish.

Utilization methods: drs, drops; h, hungry; mh, morning hungry; n, night; tgs, tea glasses; ts, teaspoon; 1 × 1, once a day; 1 × (1)2, once a day or twice a day; 1 × 2, twice a day; 1 × (2)3, twice a day or three times a day; 1 × 3, three times a day; 1 × 3 (4), three times a day or four times a day; 1 × w, once a week.

A total of 74 (78.72%) wild species and 20 (21.27%) cultivated species were recorded. Most taxa in this study were herbs (65), although there were a considerable number of shrubs (15 taxa) and trees (12 taxa). In addition, the use of one fern and one lichen were recorded in the study area.

We found that some wild medicinal plant taxa were widely used for commercial purposes. *Celtis tournefortii* (gengeres, taew, taav), *Pistacia palaestina* (bıttım, qezwan), *Pistacia eurycarpa* (bıttım, qezwan, benik), *Quercus brantii* (balot, baru beru), *Rhus coriaria* (sumaq, sımaq, tirş), *Rosa canina* (gulşîlan, gulşilav), *Thymbra sintenisii* (zahter, cahter, catir), *Thymbra spicata* (zahter, cahter), and *Urtica dioica* (gezgezok, gezo, gezgezk, qırrez) were the taxa extensively collected and marketed in the local bazaar and shops. These plants are collected mostly by women and provide a minor source of income for the local populace.

Several medicinal plants were reported as poisonous. It was explained to us that the latex should not come into contact with the tongue while using the latex of *Euphorbia craspedia*, *Euphorbia macroclada,* and *Ficus carica* species for amelioration of toothache. Also, it has been mentioned that *Teucrium polium* plant should be used in very small quantities, since an excess intake of it may cause irritation to the stomach. In addition, the hallucinogenic effect of *Hyoscyamus albus* leaves and the poisonous effect of *Dioscorea communis* roots were stated.

### Parts of Plant Used

**FIGURE 7:**
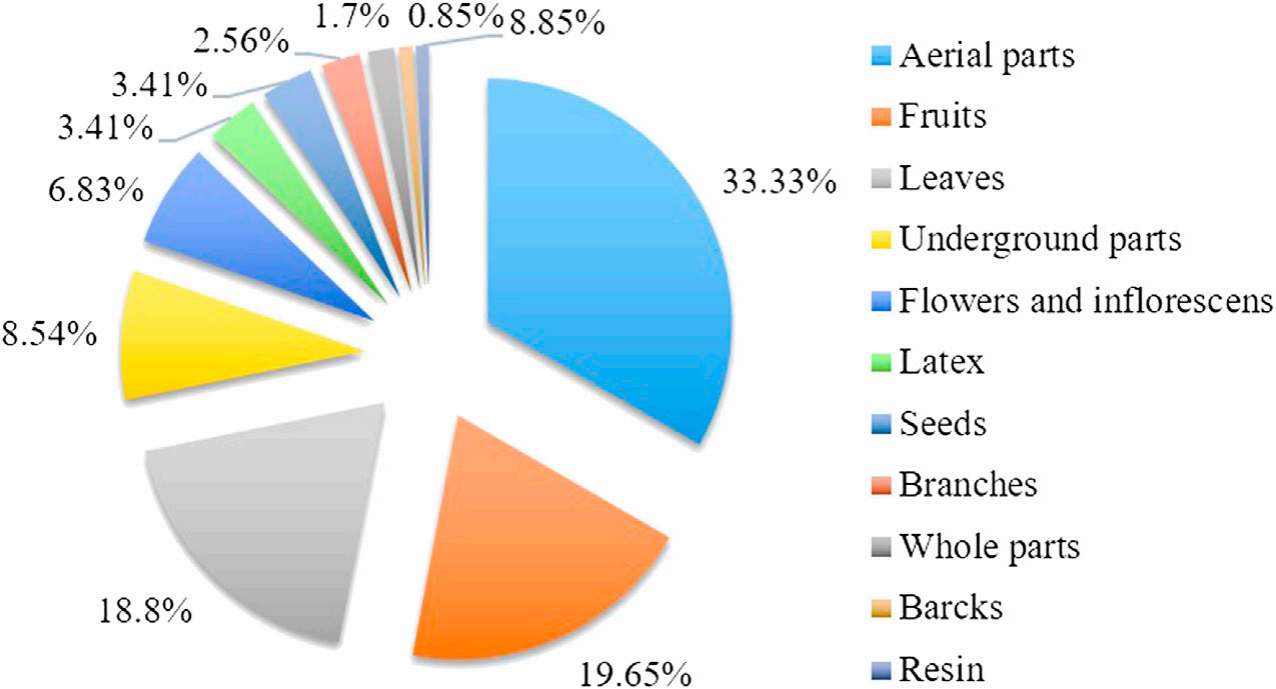
The percentages of most used plant parts in Hasankeyf.

**FIGURE 8:**
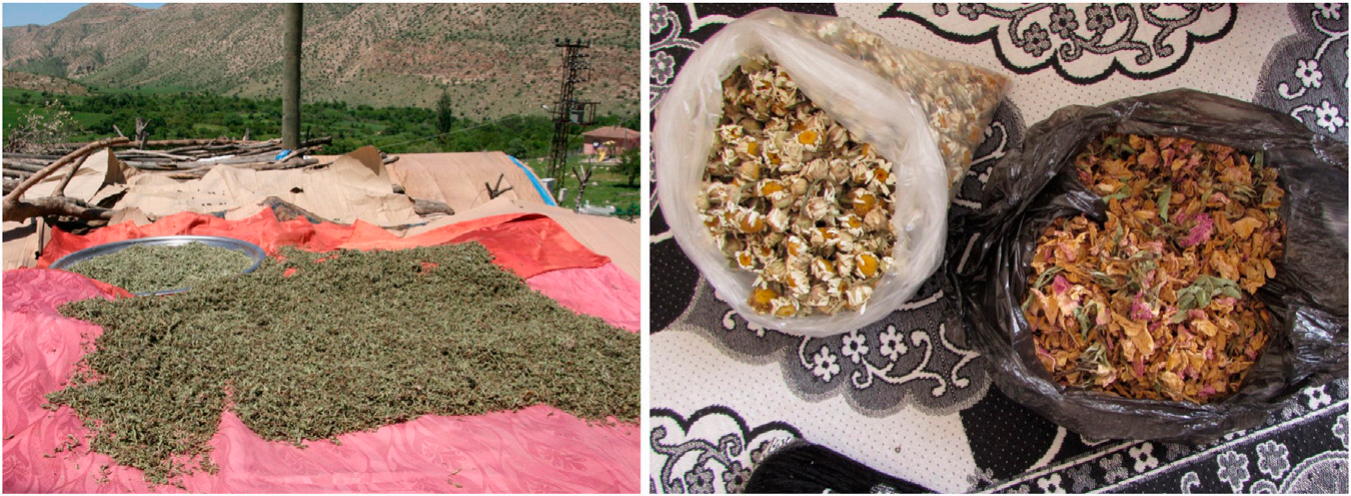
Some medicinal plants used as dried in Hasankeyf.

The aerial parts (39) of medicinal parts were the most frequently used parts in herbal drugs to cure diseases; however, many other parts were also utilized: fruits (23), leaves (22), underground parts (10), flowers and inflorescences (8), latex (4), seeds (4), branches (3), whole parts (2), barks (1), and resin (1) (Figures 7, 8). Sometimes, the local people also include other ingredients such as olive oil, animal milk, breast milk, honey, soap, salt, and sugar while preparing the remedies. In many cases, more than one organ of the same species is used in the preparation of different remedies.

### Methods of Drug Preparation and Utilization

Various forms of drug preparation include infusion, decoction, fresh application, powdering, direct, chewing, crushing, paste, cooking, chewing, sucking, burning, maceration, and molasses. The most used forms of preparation of remedies are infusion (42), fresh application (25), crushing (16), powdering (10), and decoction (13). Also, the mostly utilized administration methods for preparations are internal (55), external (31), and eaten (22). Some species used for the treatment of earache were frequently mentioned. The powdered form of the plant is often mixed with breast milk, water, or soap and applied as ear drops. During the interviews, the informants stated specifically that the milk of a mother who had bore a baby girl should be used. There are different and interesting utilization methods for ear problems. For instance, the bulbs of *Allium cepa* are crushed and the obtained juice is mixed with soap and then applied as ear drops, or the fruits of *Dioscorea communis* are dried, powdered, and mixed with water and a teaspoon of it is applied as ear drops. Another utilization method is mixing the plant material with breast milk. As an example, the seeds of *Persica vulgaris* are powdered and mixed with breast milk before being applied as ear drops (three drops, two times a day until healed); the seeds of *Hyoscyamus albus* are powdered and mixed with breast milk before being applied as ear drops. Also, the fruits of *Solanum americanum* are dried, powdered, and burnt, and the affected ear is exposed to the smoke.

Some mixtures are prepared for treatment. For instance, garlic, oil, and salt are mixed together and applied on the eyelid for alleviation of eye pain; henna and eggs are mixed together and applied to heal broken bones. Various uses of *Matricaria aurea* were recorded. It is mixed with *Urtica* species and *Malva neglecta* to treat abdominal pain. Furthermore, the infusion of *Matricaria aurea*, *Thymbra* species, and *Thymus* species is used internally for the alleviation of menstrual pain and afterpains. *Mentha longifolia* has another use apart from the internal usage of aerial parts infusion. While this infusion is hot, it is poured into a basin and childless women stand on it for a while in a sitting position. This method is used to treat women with inflammation. In addition, a mixture prepared with lemon juice, egg, and olive oil is used internally for the passing of kidney stones.

Crushed garlic bulbs are directly applied to previously blooded ringworm areas to treat ringworm.

### Ailments Treated by Plants

**FIGURE 9:**
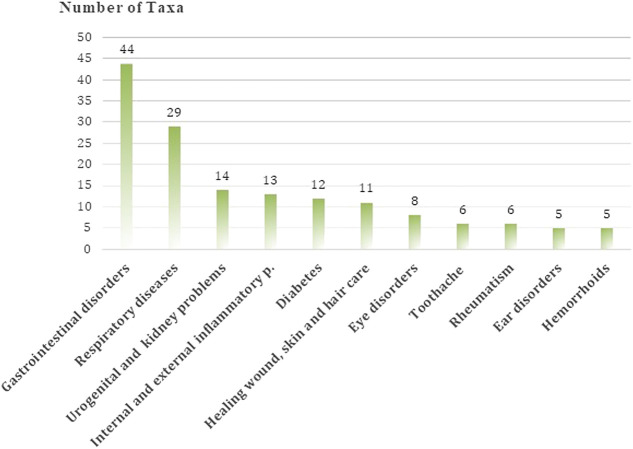
The most common ailments treated by plants in Hasankeyf.

The medicinal plants of Hasankeyf are used in the treatment of 69 different types of human ailments and diseases. Local people use herbal remedies most frequently for the treatment of gastrointestinal disorders (44), respiratory diseases (29), urogenital and kidney problems (14), internal and external inflammatory problems (13), diabetes (12), and to heal wounds, as well as for skin and hair care (11) (Figure 9).

### Calculations

Ailments were grouped into 17 categories based on information gathered from the interviews (Table 3). Internal and external inflammatory problems had the highest FIC score (0.89). *Allium cepa*, *Hyoscyamus albus*, *Plantago major,* and *Ranunculus* species were among the plant remedies indicated for these problems*.* Diabetes was recorded to have the second highest FIC (0.82). *Quercus brantii*, *Teucrium polium*, *Teucrium chamaedrys,* and *Bryonia aspera* were among the plant remedies indicated for this use. Urogenital and kidney problems were recorded to have the third highest FIC (0.79), while respiratory diseases were recorded to have the fourth highest FIC value (0.76). Rheumatic pain was ranked as the fifth ailment, with an FIC value of 0.66. An FIC value of 0.64 was recorded for ear disorders (Table 1).

According to UV analysis, species with the highest count of UV were *Teucrium polium* (0.47), *Matricaria aurea* (0.44), *Urtica dioica* (0.39), *Mentha longifolia* (0.37), *Quercus libani* (0.35), *Allium cepa* (0.29), *Alcea setosa* (0.28), *Malva neglecta* (0.25), *Hypericum triquetrifolium* (0.25), *Anchusa strigosa* (0.24), *Rhus coriaria* (0.23), *Lepraria finkii* (0.23), *Anchusa azurea* (0.22), and *Tribulus terrestris* (0.22) (Table 3).

## Discussion

### Consumption of Medicinal Plants as Food

A large proportion of medicinal plants are also being used as food in the region, thus indicating that the use of wild plants has a high potential in the area. About 49% (46 taxa) of the recorded medicinal plants in Hasankeyf are consumed in various ways as food. The wild medicinal plants investigated in this study are quite widely consumed raw as salad, pickles, preserves, and fruits. Also, they can be boiled and fried in a meal. *Anchusa azurea*, *Anchusa strigosa*, *Malva neglecta*, *Nasturtium officinale*, *Urtica dioica*, *Urtica pilulifera*, *Papaver glaucum*, *Punica granatum*, *Quercus brantii*, *Rubus sanctus*, *Allium kharputens*, *Allium ampeloprasum*, *Allium scorodoprasum,* and *Celtis tournefortii* were the medicinal plants most consumed as food in Hasankeyf.

Moreover, some medicinal plants were used as spices, especially in the rural areas. *Cyclotrichium leucotrichum*, *Mentha longifolia* subsp. typhoides, *Origanum vulgare* subsp. gracile, *Rhus coriaria*, *Thymbra sintenisii*, *Thymbra spicata,* and *Thymus kotschyanus* were used as spices.

In the study area, some medicinal plants were used as herbal tea, such as *Melissa officinalis*, *Salvia multicaulis*, and *Thymbra spicata.* In addition, *Pistacia palaestina* was consumed as coffee (Yeşil and İnal, 2019).

Since *Arum rupicola* is known for its toxic nature in the study area, local people subject it to a mandatory detoxification process before consuming it as medicine and food. This detoxification process has also been noted in previous ethnobotanical studies (Yeşil and Akalın, 2010; Pieroni et al., 2017; Pieroni et al., 2018; Yeşil and İnal, 2019; Yeşil et al., 2019; Kılıç et al., 2020).

In the study area, a syrup named “Gezo” was obtained from the leaves of the *Quercus* species by immersing them in a warm water spring. Thus, the concentrated sweet substance on the surface of the leaf is transferred into water. The obtained syrup is consumed as food when cooled and is also used as medicine for boosting the immune system; for treating cardiac diseases, stomach problems, diabetes; and for healing wounds. Furthermore, this syrup is added to molasses of grape and used for boosting the immune system. It is believed that Gezo fell down from the sky on oak trees, especially on to its leaves. It is produced by parasites of oak trees. The consumption of Gezo as food was stated previously by Gençay (2007) and Behçet and Arık (2013). Moreover, polyphenols analyzed by UHPLC-ESIMS/MS and antioxidant activities of molasses, acorn, and leaves of oak were studied, and it was found that Gezo molasses demonstrated higher scavenging effects than other extracts (Bursal et al., 2018).

There are more than 200 *Allium* species naturally distributed in Turkey and 30 of them have been used widely by the local people for various purposes as vegetable, spice, condiment, and medicine (Ekşi et al., 2020). In this study, it can be seen that five *Allium* taxa are used for medicinal purposes by the local people.

### Review of Local Plant Names

The study area and the surrounding provinces comprise a multilingual population. When the plant names are generally examined, it was found that most of the plant names were in Kurdish and Arabic; only a few plant names were in Turkish.

Kozluk (Batman), Cizre (Şırnak), Artuklu (Mardin), and Midyat (Mardin) are close to the study area. Daisies are generally called *beybun* or *beybanuç* in the region. One of the most used plants *Matricaria aurea* is called *giyayêseva*, *gihaseva*, which means “apple herb” in Hasankeyf. The feature that distinguishes this plant from other daisies is the apple (“Sev” in Kurdish) scent of the plant. The plant is also known by the same name in Artuklu (Mardin) (Kılıç et al., 2020) and Midyat (Mardin) (Akgül et al., 2018).

On the other hand, the names of some local plants used in these areas are different. These local plants include *Anchusa strigosa* (hımhım), *Cydonia oblonga* (verekılfercel), *Hypericum triquetrifolium* (aran, ğırsile), *Juniperus oxycedrus* (dıfran), *Malva neglecta* (hıbbes, tıbbayka, tabaknunu), *Paliurus spina-christi* (mağaylun), *Salvia multicaulis* (baravine, ikoro), *Teucrium polium* (cığde), and *Tribulus terrestris* (pıruğacuz) in Midyat (Mardin) (Akgül et al., 2018); *Achillea aleppica* (Kulilkamera, Isfaysara), *Echinops orientalis* (şekerok, şekirok), *Melissa officinalis* (pung, rihıtınneebune, nınhe), *Salvia multicaulis* (çaya çiyan, ikoro, bızzeyn), *Salvia palaestina* (çaya çiyan, ikoro, bızzeyn), *Paliurus spina-christi* (driya çalo, hezisk, mığeylen, sınc, selunê), and *Tribulus terrestris* (kurincok, korincok) in Artuklu (Midyat (Kılıç et al., 2020); *Cyclotrichium leucotrichus* (punge tata), *Plantago major* (belgeves), *Salvia multicaulis* (kaşketin), *Salvia palaestina* (ada çayı), *Teucrium polium* (merwend), and *Tribulus terrestris* (gurnig, kartıba) in Kozluk (Batman) (Bulut et al., 2019); and *Prosopis farcta* (hışhaş), *Hypericum triquetrifolium* (kantaron), *Salvia multicaulis* (giyacılık), and *Teucrium chamaedrys* (bojdank) in Cizre (Şırnak) (Gençay, 2007).

### Comparison of the Obtained Data With Those of Nearby Regions

We calculated the similarity index of our study against other comprehensive studies conducted in nearby areas (Gençay, 2007; Yeşil and Akalın, 2009; Çakılcıoğlu and Türkoğlu, 2010; Tuzlacı and Doğan, 2010; Çakılcıoğlu et al., 2011; Polat et al., 2013; Kaval et al., 2014; Akgül et al., 2018; Polat, 2019; Kaya et al., 2020; Kılıç et al., 2020) and short-term ethnobotanical study of plants used for folk medicine in a nearby area (Bulut et al., 2019). The similarity index varied from 16.92 to 46.87%. Maximum similarity was observed in the ethnobotanical study of plants in Midyat (Mardin). The first factor in this similarity is that Midyat is a close neighbor of Hasankeyf, and the second is they have been sharing similar habitats, flora, and similar social structures. The lowest index was obtained for Çatak (Van) (Mükemre et al., 2015), which was probably caused by the differences due to floral diversity and the characteristic habits of Çatak.

When we reviewed the plant families used for medicinal purposes in our study, Lamiaceae family emerged first. However, the Lamiaceae family emerged in first place only in the study conducted in Urfa (Kaya et al., 2020), while Asteraceae emerged first in all other studies compared. Furthermore, unlike other regions, we observed that plants in the Amaryllidaceae family are frequently used for various medicinal purposes in Hasankeyf.

In addition, the taxa not included in the studies presented in Table 4 are as follows *Alcea digitata*, *Allium kharputense*, *Althaea cannabina*, *Dioscorea communis*, *Euphorbia craspedia*, *Hyoscyamus albus*, *Papaver glaucum*, *Ranunculus millefolius*, and *Scandix stellata*. The medicinal purpose of these taxa were first recorded by this study in Hasankeyf and its surroundings.

**TABLE 4:** The similarity percentages of studies in nearby areas.

Citation	Location	Number of informants	Total medicinal taxa	Common medicinal taxa	Similarity percentage (%)
Akgül et al. (2018)	Midyat (Mardin)	123	32	15	46.87
Çakılcıoğlu and Türkoğlu (2010)	Sivrice (Elazığ)	176	81	21	25.92
Çakılcıoğlu et al. (2011)	Maden (Elazığ)	143	88	21	23.86
Gençay (2007)	Cizre (Sırnak)	60	44	19	43.18
Kaval et al. (2014)	Geçitli (Hakkâri)	146	70	12	17.14
Kaya et al. (2020)	Urfa	195	37	14	37.73
Kılıç et al. (2020)	Artuklu (Mardin)	365	85	32	37.64
Polat et al. (2013)	Solhan (Bingöl)	145	82	25	30.48
Polat (2019)	Bingöl	182	93	24	25.80
Tuzlacı and Doğan (2010)	Ovacık (Tunceli)	—	65	11	16.92
Yeşil and Akalın (2009)	Kürecik (Malatya)	120	47	13	27.65

In this study, it was observed that four of the five *Allium* species used for medicinal purposes in Hasankeyf were also used for the treatment of the ailments of the eye by the local populace. Furthermore, the use of *Allium kharputense* as food has been noted in previous studies (Gençay, 2007; Yeşil and Akalın, 2010; Arı et al., 2015; Fırat, 2015; Yeşil and İnal, 2019). However, its medicinal use (for the treatment of eye pain) is recorded for the first time in Hasankeyf by this study. Antibacterial and antimicrobial activities of *Allium* species were stated in several studies (Fritsch and Keusgen, 2006; Panomket et al., 2012). Also, antimicrobial effect of methanolic extract from *Allium kharputense* has been previously determined (Erdoğan et al., 2015; İzol et al., 2020).

Medicinal use of plants from the Solanaceae family was not observed in ethnobotanical studies conducted in the regions close to Hasankeyf (Gençay, 2007; Polat, 2019; Yeşil and Akalın, 2009; Çakılcıoğlu and Türkoğlu, 2010; Tuzlacı and Doğan, 2010; Çakılcıoğlu et al., 2011; Polat et al., 2013; Kaval et al., 2014; Akgül et al., 2018; Bulut et al., 2019; Kaya et al., 2020; Kılıç et al., 2020). The use of tobacco was recorded by Gençay (2007) for pleasure. Since its use is common, it may not be included in other studies. In addition, medicinal use of some members of the Solanaceae family is known in the eastern Anatolian region (Altundağ and Öztürk, 2011). Nevertheless, *Solanum americanum* has never been known for medicinal use in the East and Southeast of Turkey. In the study area, the smoke of burnt fruit of *Solanum americanum* is used for curing ear problems. As mentioned in a recent study (Afroz et al., 2020), *Solanum* genus contains plenty of snakin-2 peptide, which has significant antimicrobial activity. Also, there are four more taxa used for ear problems: *Allium cepa*, *Dioscorea communis*, *Hyoscyamus albus*, and *Persica vulgaris*. However, *Dioscorea communis*, *Hyoscyamus albus*, and *Persica vulgaris* were not mentioned in previous studies conducted in Eastern Turkey.

When this study is compared with other studies in terms of the number of informants and the number of medicinal taxa, it is clearly seen that the residents of Hasankeyf use traditional knowledge more efficiently. The reasons for this may be its long-standing background, its multicultural population, and its inhabitants’ being in touch with nature and the diversity of flora.

**FIGURE 10:**
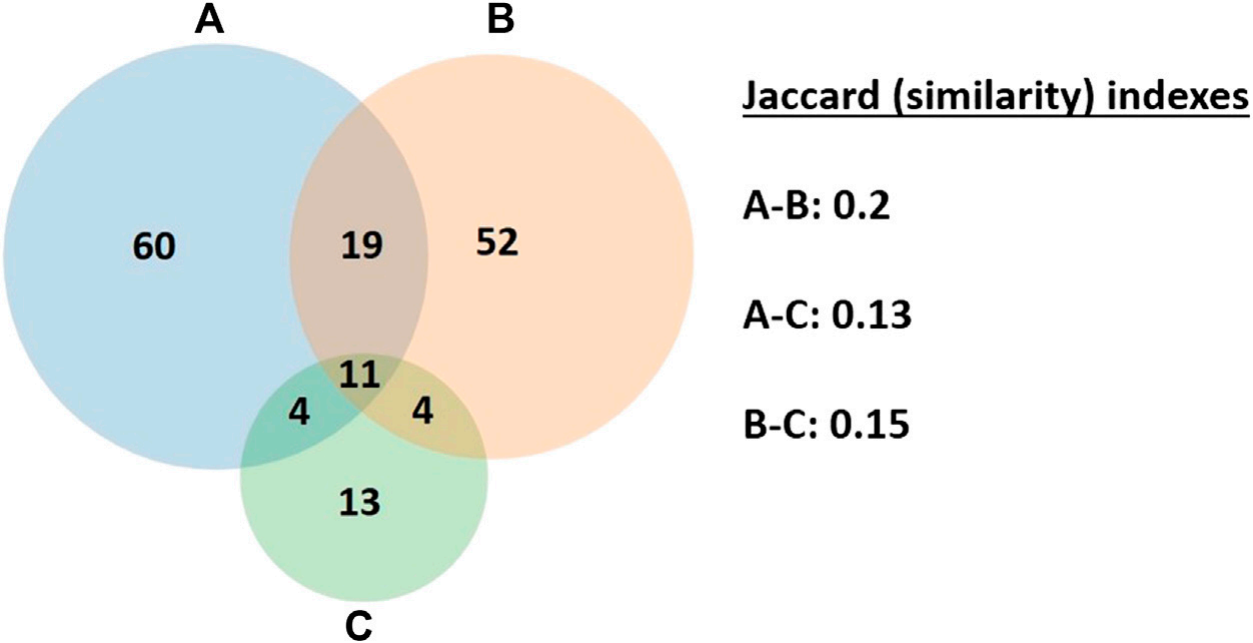
The overlap in the medicinal plants by three close regions: **(A)** Hasankeyf (Batman); **(B)** Artuklu (Mardin); **(C)** Midyat (Mardin).

A Venn diagram showing the overlap among Hasankeyf and two nearby regions (number of cited taxa) with high similarity percentages and the related Jaccard Similarity Indexes are presented in Figure 10. The most overlap of the obtained data and the Jaccard index was between Hasankeyf and Artuklu (Kılıç et al., 2020), which may be due to the similarity of the density of Kurdish and Arab populations (Muslims) in Artuklu and Hasankeyf. The least overlap, on the other hand, was between Hasankeyf and Midyat (Akgül et al., 2018), and the major reason for that seems to be that Midyat has a multireligious population. Ten taxa are used in common in all three regions: *Anchusa azurea*, *Capparis sicula*, *Hypericum triquetrifolium*, *Malva neglecta*, *Matricaria aurea*, *Teucrium polium*, *Thymbra sintenisii*, *Tribulus terrestris*, *Paliurus spina-christi*, and *Urtica dioica*. These taxa are culturally important plants in all three regions as they are used for different purposes (food, ornament, fuel, etc.) besides their medicinal use.

The medicinal use of *Matricaria aurea* species was only recorded in Hasankeyf, Midyat (Mardin) (Akgül et al., 2018), and Artuklu (Mardin) (Kılıç et al., 2020) regions. This species was the second plant with the highest UV in Hasankeyf and was frequently mentioned by women. In a previous study, it was stated that essential oils of *Matricaria aurea* are very rich in chemical compounds, especially phenolic-containing coumarin products, which have contributed to their antioxidant and antibacterial activity (Kheder et al., 2014).

The use of fruits of *Paliurus spina-christi* for toothache has been previously recorded (Tuzlacı, 2016). However, the use of the greasy material (obtained by burning the branches of the plant) for toothache was recorded for the first time in this study.

It was mentioned that the inflorescences of the *Echinops orientalis* plant directly struck the body to eliminate excess blood (bloodletting). Bloodletting, the practice of letting blood out to cure a patient, was for centuries one of the main therapies in the West (Miton et al., 2015). The use of *E. orientalis* for an ancient treatment method shows that the residents of Hasankeyf had impressive traditional knowledge.

## Conclusion

In this study, the use of 94 plant taxa for 69 different types of ailments and diseases was determined in the Hasankeyf district. As a result of interviews with the individuals, it was observed that the plants frequently recorded in questionnaires include *Teucrium polium*, *Matricaria aurea*, *Urtica dioica*, *Mentha longifolia Quercus brantii*, *Allium cepa*, *Alcea setosa*, *Malva neglecta*, *Hypericum triquetrifolium*, *Anchusa strigosa*, *Rhus coriaria*, *Lepraria finkii*, *Anchusa azurea,* and *Tribulus terrestris*. These plants also have a widespread usage in the area and higher UVs. Also, the data indicate that the main illnesses treated by medicinal plants in Hasankeyf are gastrointestinal disorders, respiratory diseases, urogenital and kidney problems, internal and external inflammatory problems, diabetes, healing of wounds, as well as skin and hair care. Moreover, the treatment of ear problems, of which there are few records in ethnobotanical studies, has been frequently mentioned in the study area, and the uses and use methods of plants are stated by both young and old individuals. In addition, the method of bloodletting by the spins of a plant, which has an ancient history in the study area, is not recorded in ethnobotanical studies in the East and Southeast regions of Turkey. Taxa such as *Allium kharputense*, *Althaea cannabina*, *Dioscorea communis*, *Euphorbia craspedia*, *Hyoscyamus albus*, *Papaver glaucum*, *Ranunculus millefolius,* and *Scandix stellata* were not recorded in nearby areas before.

It was observed that individuals living in rural areas have more traditional knowledge about the use of medicinal plants than those living in urban areas. In addition, it was observed that women were more experienced in using plants compared to men; for instance, women aged 51 and over had knowledge about preparation and utilization methods of medicinal herbs in rural areas. Since the older individuals living in villages and individuals who have traditional knowledge are not educated, they speak only Kurdish; thus, they are not exposed to both TV and social networks (Kurdish is not used commonly in these mediums). However, this disadvantage turned into an advantage because such traditional knowledge is not contaminated with external information and is therefore undilutedly passed on from generation to generation. On the other hand, the knowledge is transmitted orally, which may lead to loss or at least deformation of information by time. At this point, this study is of great importance as this valuable knowledge is recorded.

Although, men in urban areas generally had knowledge about medicinal plants, detailed plant uses were mostly explained by women. Moreover, it has been observed that individuals in urban areas aged 61 and over had knowledge about medicinal plants. It is inevitable that the traditional use of plants will rapidly disappear from the cities and some villages due to migration to modern centers where lifestyles are completely different (livelihood, gathering food, receiving health care, new people, TV, language, etc.)

The obtained traditional knowledge is an important resource for sustainable development in the study area. In particular, by establishing small-scale local cooperatives, it can be ensured that commonly used medicinal plants (*Matricaria aurea*, *Hypericum triquetrifolium*, *Salvia multicaulis*) are produced under suitable conditions and sold in the public market or in local shops in the region. In addition, some culturally important plants (*Anchusa* speies, *Glycyrrhiza glabra*, *Pistacia palaestina*, *Thymbra* species) can be grown in the region and these can be prepared and served in local restaurants using traditional methods. Furthermore, in the light of the obtained knowledge, it was observed that there were some plants that can be used to boost the immune system and to treat symptoms similar to that of Covid-19. However, as there are no enough previous studies, the chemical contents and activities of these plants should be investigated in detail.

Another significance of this study is that the obtained data may be an important and meaningful source for future ethnobotanical studies in Southeastern Turkey, as there have been very few studies carried out on plants due to local problems encountered in the region.

## Data Availability

The raw data supporting the conclusions of this article will be made available by the authors, without undue reservation, to any qualified researcher.

## Appendix A

1.   Name and surname of the participant
2.   Age and sex of the participant
3.   Telephone and address of the participant
4.   Educational level of the participant
5.   Date of interview
6.   Place of residence of the participant
7.   Duration of residence of the participant
8.   What is the local name of the plant used?
9.   For which diseases do you use the plant?
10. Which parts of the plant do you use (root, stem, flower, leaves, fruit, etc.)?
11. How do you prepare the plant for use?
12. How and when do you use the plant?
13. Which group of age can use the plant?
14. Approximately what dose do you use?
15. How long does the convalescence period take?
16. Did any complication occur from the plants you used?
